# Auditory Feedback for Enhanced Sense of Agency in Shared Control

**DOI:** 10.3390/s22249779

**Published:** 2022-12-13

**Authors:** Tomoya Morita, Yaonan Zhu, Tadayoshi Aoyama, Masaru Takeuchi, Kento Yamamoto, Yasuhisa Hasegawa

**Affiliations:** Department of Micro-Nano Mechanical Science and Engineering, Nagoya University, Nagoya 464-8601, Japan

**Keywords:** robotic teleoperation, sense of agency, auditory feedback

## Abstract

There is a growing need for robots that can be remotely controlled to perform tasks of one’s own choice. However, the SoA (Sense of Agency: the sense of recognizing that the motion of an observed object is caused by oneself) is reduced because the subject of the robot motion is identified as external due to shared control. To address this issue, we aimed to suppress the decline in SoA by presenting auditory feedback that aims to blur the distinction between self and others. We performed the tracking task in a virtual environment under four different auditory feedback conditions, with varying levels of automation to manipulate the virtual robot gripper. Experimental results showed that the proposed auditory feedback suppressed the decrease in the SoA at a medium level of automation. It is suggested that our proposed auditory feedback could blur the distinction between self and others, and that the operator attributes the subject of the motion of the manipulated object to himself.

## 1. Introduction

For many years, research has been conducted on operational robots that work remotely in place of humans in extreme environments such as nuclear power plants, undersea, and at fire scenes [1]. In recent years, there has been a growing need for robots that can be operated remotely, such as by blue-collar teleworkers. In robotic teleoperation, a sense of immersion in the workspace and operability are major factors that contribute to the achievement of aims. Our research group have developed an intuitive system that enables immersive and intuitive robot teleoperation [2,3]. In this system, the position and posture of the VR controller and those of the head-mounted display (HMD) worn by the operator can be reflected on the arm and head of the remote robot, respectively, and parallax images from the stereo camera mounted on the robot head are displayed on the HMD. This kind of immersive robot teleoperation can be expected to further improve operability by promoting robotic embodiment. Robotic embodiment is the illusion that the operator perceives the robot arm or robot body as part of his or her own, which allows the operator to plan and control the robot’s motion as if it were his or her own body [4,5,6,7,8,9,10,11]. One of the factors that promote robotic embodiment is sense of agency (SoA). SoA refers to the sense of recognizing that the motion of an observed object is caused by oneself [12], and it has been attracting attention in the field of rehabilitation in recent years [13].

SoA decreases when the movement of the manipulated object differs from the operator’s intention. This can be explained by the comparator model, which has been proposed as a mechanism for generating SoA [12,14,15]. SoA arises when the sensory feedback predicted by the internal prediction model formed in a human brain matches the perceived actual sensory feedback. Conversely, when the actual feedback differs from the intended feedback, the SoA decreases. This decrease is expected to cause a decrease in motivation for the task [16].

Therefore, maintaining a high SoA is also important for robot teleoperation, and it is necessary to reflect the operator’s intention in the robot’s movements to prevent a decline in SoA.

**Figure 1:**
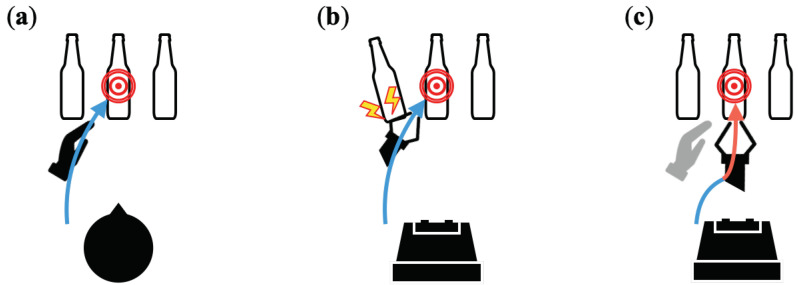
Shared control of grasping assistance to resolve differences in human and robot physical structures: (**a**) Humans plan movements based on information about their own physical structure. (**b**) When robots are manually and remotely operated by humans, differences in the physical structures of humans and robots reduce operability. (**c**) Shared control, which fuses control commands from humans and robots, supports task execution.

However, it would be extremely difficult, both from the standpoint of safety and the burden of operation, for a person to remotely control the entirety of the robot’s degree of freedom while knowing in advance the physical characteristics of the work environment, including not only the size and shape of the robot arm but also the robot’s structure (e.g., range of motion of joints). Time delay is also a common issue in robotic teleoperation and significantly impairs operability [17]. Therefore, shared control, in which the robot executes tasks by merging operation commands from humans and operation commands planned by the robot itself based on its own judgment, is expected to reduce the workload and improve safety. This enables both real-time operation in which the robot makes decisions and acts on its own based on a large amount of real-time information obtained from the work site, and intelligent operation based on advanced cognition and judgment by the operator based on the received information [18,19]. This is a valuable method for the operation of robots that handle complex environments and objects, such as construction machinery operation [20] and surgery [21]. In addition, as shown in Figure 1, when we operate robots remotely, differences in physical structures between humans and robots greatly reduce the operability and transparency of the system, making it difficult to perform accurate operations and avoid hazards at the work site. Therefore, shared control, which grants more authority to the robot, may be required to ensure that the robot performs its tasks and avoids obstacles. In this study, we focused on shared control in terms of the reaching motion when grasping an object.

In the teleoperation of robots, it is desirable for a person to operate a robot based on recognition and judgment in a state of enhanced embodiment, and for the robot to make judgments in real time to provide work support and avoid danger. To achieve this, it is necessary to realize shared control that does not diminish the operator’s SoA. The findings of this paper are as follows:

1 Increasing the level of automation by shared control for robot manipulation decreased the SoA and increased task performance;
2 At a moderate level of automation, auditory feedback linked to both the operator’s manipulations and the robot’s movements suppressed the decline in the SoA.

## 2. Related Works

Recent studies have reported that both internal motor signals and external cues contribute to SoA, and that external cues play an important role in judgments of agency in ambiguous situations [22]. This cue-integration theory explains that the SoA is generated by integrating multiple cues, and that the weighting is determined by the reliability of each cue. Wen et al. conducted an experiment in which participants manipulated the direction of a moving dot on a display using a keyboard and evaluated their SoA by changing the presence or absence of an assistant and the time delay for manipulation [23]. They reported that when the action–feedback association was uncertain, participants felt a higher SoA when the task performance, one of the external cues, was higher. Ueda et al. experimented with a task in which participants manipulated the left–right movement of a cursor on a display using a joystick and followed a target that moved irregularly to the left and right by gradually changing the level of automation, and they investigated the relationship between the level of automation, SoA, and task performance [24]. In this experiment, subjective evaluations of the SoA and performance increased with increasing levels of automation when the level of automation was within a certain range. However, the SoA decreased when the level of automation exceeded a certain range, even if the performance evaluation increased. Aoyagi et al. also reported that feedback that visually corrects the position of manipulated objects in virtual space, which is controlled by the operator’s hand position, improved the SoA [25].

As shown in these previous studies, the SoA may be maintained or enhanced even when intervention by others to assist manipulation occurs and the operator’s own manipulations do not exactly match the object’s movements. In this study, we focused on auditory feedback, which is one of the external cues, and aimed to solve the problem of the difficulty of synchronizing subjective operation with operation support by presenting auditory feedback that aims to blur the judgment of the distinction between self and others with respect to the subject of the action. Specifically, we aimed to increase the reliability of auditory feedback during integration by presenting auditory feedback modified to better reflect one’s own operation during shared control, blurring the distinction between self and others with respect to the operation, and preventing SoA from diminishing. This method is inspired by previous research in which the operator attributes the subject to himself in situations where the subject of the manipulated object’s action is ambiguous. Although there have been many studies examining the effects of time delays [26,27,28,29,30] and manipulation interventions from others [24,31] on SoA, very few studies aiming to reduce their effects on the SoA by auditory feedback are available. Furthermore, most of the studies on SoA have dealt with the manipulation of objects on a 2D plane or a single action, and it is questionable whether they can be applied to robot manipulation in which the position and posture of an arm are continuously manipulated in 3D space. Therefore, we constructed a VR environment in which the position and posture of a robot arm can be continuously manipulated in a 3D space and auditory stimuli can be presented to the operator and during the conducted experiments.

## 3. Auditory Feedback and Experiments

We constructed a VR simulator for the intuitive teleoperation system in which a robot in a remote location is controlled by VR devices from a first-person perspective. In this simulator, shared control, which continuously supports the positioning and posture determination operations of the virtual robot gripper, was introduced, and experiments were conducted to track a regularly moving target object.

### 3.1. Experimental Setup

**Figure 2:**
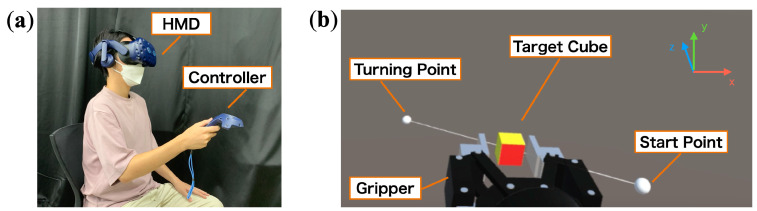
Experimental setup: (**a**) The operator wears the HMD while sitting on a chair and holds the controller in his right hand. (**b**) An image of the operator’s viewpoint displayed on the HMD.

The main component of this study is a VR-based HMD (HTC VIVE Pro Eye, HTC Corporation). The HMD is wired to a Windows PC (Intel Core i7-6700K 4.00 GHz, 16 GB RAM) with a game engine (Unity 2020.3.36f1., Unity Technologies) installed and can be used within the virtual space created by Unity using the SteamVR Plugin version 2.7.3. The virtual robot gripper and the target object, a 2 cm cube, are placed in a virtual space created by Unity (Figure 2).

The position of the gripper ($x_{gripper}$) and its rotation around the *z*-axis ($\theta_{z\_gripper}$) are controlled according to the following equations:

(1) $$x_{gripper}=\left(1-\alpha \right)\cdot x_{controller}+\alpha \cdot x_{cube},$$

(2) $$\theta_{z\_gripper}=\left(1-\alpha \right)\cdot \theta_{z\_controller}+\alpha \cdot \theta_{z\_cube}.$$

**Figure 3:**
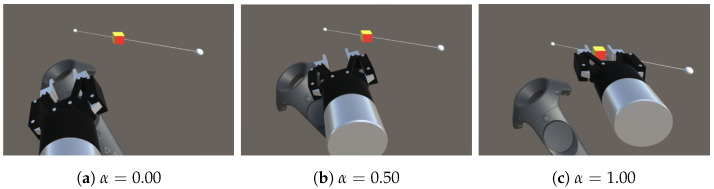
Position and orientation relationships between controller, virtual gripper, and target cube: (**a**) The gripper synchronizes with the controller. (**b**) The position and orientation of the gripper are between the controller and the target cube. (**c**) The gripper synchronizes with the target cube. In these figures, the controller model is shown for comparison but was not used during the experiment.

As shown in Figure 3, by changing the automation ratio α (0.0≤α≤1.0), the intensity of the manipulation intervention can be changed between manual control (α=0.00), which is synchronized with the position ($x_{controller}$) and rotation ($\theta_{z\_controller}$) of the controller, and fully automatic control (α=1.00), which is synchronized with the position ($x_{cube}$) and rotation ($\theta_{z\_cube}$) of the target object. In this experiment, six levels of automation are presented (α= 0.00, 0.25, 0.50, 0.75, 0.90, and 1.00).

**Figure 4:**
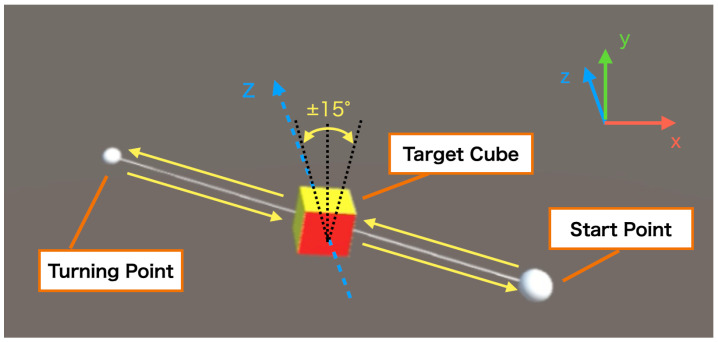
Movement of the target cube: The target cube makes one round trip on a line segment connecting two different points (start point and turning point) in virtual space, taking approximately 15 s. At the same time, the cube repeatedly rotates $\pm 15^{\circ }$ around the *z*-axis in 2 s per round trip.

The position of the target object ($x_{cube}$) is controlled according to the following equations:

(3) $$x_{cube}=\left(1-\beta \right)\cdot x_{start}+\beta \cdot x_{turn},$$

(4) $$\beta =\frac{1}{2}\left(\sin\left(K\cdot t-\frac{\pi }{2}\right)+1\right),$$

where $x_{start}$ and $x_{turn}$ are the positions of the start and turnaround points of the target trajectory, respectively, *t* is the elapsed time from the start of the task, and *K* is a constant. The target cube oscillates singly on the line segment connecting the two points in the virtual space. The positions of the two points are initialized with respect to the initial position coordinates of the HMD, and the distance between the two points is 15 cm, 10 cm, and 30 cm in the *x*, *y*, and *z* axes, respectively. In this experiment, it takes about 15 s for the target object to make one round trip. At the same time, the target object rotates back and forth repeatedly in the $\pm 15^{\circ }$ section around the *z*-axis. The target object rotates at a constant angular speed, making one round trip in 2 s (Figure 4).

### 3.2. Auditory Feedback

In this experiment, the following four sound conditions are used to verify the effectiveness of the proposed auditory feedback method.

None No sound presentation.Controller Present sound linked to controller speed and angular speed.Gripper Present sound linked to gripper speed and angular speed.Mixed Present mixed sound of the Controller and Gripper conditions.

In this experiment, since SoA is easily affected by the difference in velocity between the manipulated object and the hand [31], the sounds linked to velocity are presented. The sounds are generated by constantly playing a beep sound in the virtual space and changing the pitch. The velocity ($v_{sound}$), which is the composite of the controller velocity and the gripper velocity, and the angular velocity ($\omega_{sound}$), which is the composite of the controller angular velocity and the gripper angular velocity, are defined as follows:

(5) $$v_{sound}=\left(1-\gamma \right)\cdot v_{controller}+\gamma \cdot v_{gripper},$$

(6) $$\omega_{sound}=\left(1-\gamma \right)\cdot \omega_{controller}+\gamma \cdot \omega_{gripper},$$

where the value of the parameter γ changes the auditory feedback conditions (controller: γ=0.0; gripper: γ=1.0; mixed: γ=0.5). Then, the pitch of the sound (*p*) presented varies with the magnitude of $v_{sound}$ and $\omega_{sound}$, as follows:

(7) $$p=a\cdot |v_{sound}\left|+b\cdot \right|\omega_{sound}|,$$

where *a* and *b* are constants, and their values are determined empirically. The upper and lower limits for the sound pitch value are set so that the sound is in the audible range. The generated sounds are presented to the operators through headphones attached to the HMD.

### 3.3. Participants

Ten able-bodied students (male, mean age 24.7, SD=1.73) from Nagoya University participated in this experiment. All participants were right-handed and had normal or corrected vision, and they had little or no experience with using VR devices. All participants’ data are accounted for in the analysis. This experiment was approved by the Ethics Review Committee at Nagoya University; all participants were selected from outside our research group and received monetary compensation for their participation.

### 3.4. Tracking Task

**Figure 5:**
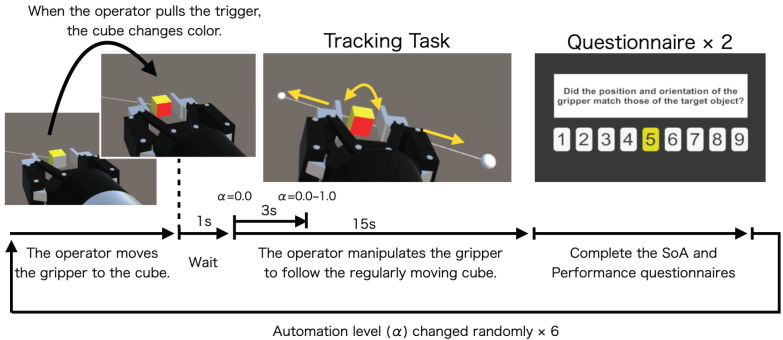
Procedure for one set of the tracking task.

Figure 5 shows the procedure for one set of the tracking task. First, the operator manipulates the position and posture of the virtual robot gripper with a VR controller and moves it to the front of the target object, which is stationary at the initial position. This alignment phase is performed for each trial, and it also contributes to the participants becoming used to the VR controller operation and promoting the SoA. The task is then started by pulling the trigger on the controller while holding the cube in the center of the gripper. At this point, the color of the front of the cube changes from gray to red, clearly indicating to the operator that the tasks have been started. The target object starts regular rotational and translational motion one second after the task starts. The operators are instructed to follow the position and posture of the target object with the gripper. To avoid the operator becoming aware of the intervention due to a sudden change in the automation level, the automation rate α is changed gradually from 0 in 3 s to a value determined randomly from among six levels. After the target cube makes one round trip between the two points, a questionnaire response screen is displayed on the HMD, and participants rate SoA and task performance on a 9-point scale from 1 to 9 after each trial. The question “Was the movement of the gripper controlled by yourself?” for SoA was followed by the question “Did the position and orientation of the gripper match those of the target object?” for task performance. These questions were modified from those used in previous studies [24,25,31] to fit this task.

### 3.5. Experimental Procedure

While sitting in a chair in a relatively quiet room, the participants put on the HMD and operated a VR controller with their right hand (Figure 2a). Before starting the experiment, participants received verbal explanations on how to operate the gripper, the contents of the task, and how to answer the questionnaire while viewing the experimenter’s viewpoint image on the monitor while the experimenter completes the tracking task and completed the questionnaire. Participants were also informed in advance that intervention from others might occur in gripper manipulation. The tracking task was repeated in a randomized order of the six automation levels, and one set was completed by answering the questionnaires after each trial. All auditory feedback conditions were divided into two sets; specifically the first half and the second half, and the order of the auditory feedback conditions was changed between the first and second half so that the order of the conditions did not affect the questionnaire results. There were breaks between the first and second halves to ensure that the tasks in the first half did not affect the results of the second half. In other words, 48 trials (6×2×4) were conducted per participant. The order of the auditory feedback presented across subjects was also changed randomly so that the order of the experiments would not affect the results. The experiment, including explanations, required approximately one hour per participant.

## 4. Results

### 4.1. Sense of Agency Rating

**Figure 6:**
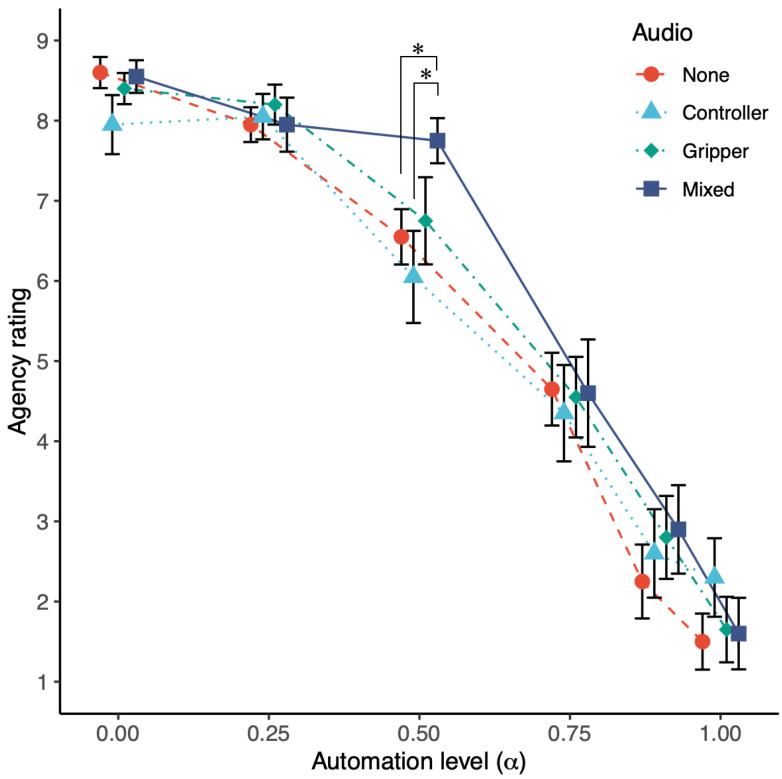
Mean agency ratings at each level of automation under four different auditory feedback conditions. Error bars represent standard errors. The SoA decreases with increasing automation level and is significantly higher in mixed condition than in none and controller condition when the automation ratio α is 0.50 (*: p<0.05).

Figure 6 shows the mean agency ratings at each level of automation under four different auditory feedback conditions. To examine the effect of automation on SoA, Friedman’s test was conducted in each auditory feedback condition. The test shows significant differences between the automation levels in all conditions (none: $\chi^{2}\left(5\right)=47.872$, p<0.01; controller: $\chi^{2}\left(5\right)=46.287$, p<0.001; gripper: $\chi^{2}\left(5\right)=46.149$, p<0.001; mixed: $\chi^{2}\left(5\right)=45.088$, p<0.001). To examine the effect of auditory feedback on SoA, tests were conducted for each automation level condition. Wilcoxon’s signed rank test was used to test the difference between the two groups, and Bonferroni correction was used for multiple comparisons. The test results indicate that, at the medium level of automation (α=0.50), the SoA is significantly higher in the mixed condition than in the none and controller conditions (p=0.034; p=0.029, respectively).

### 4.2. Task Performance Rating

**Figure 7:**
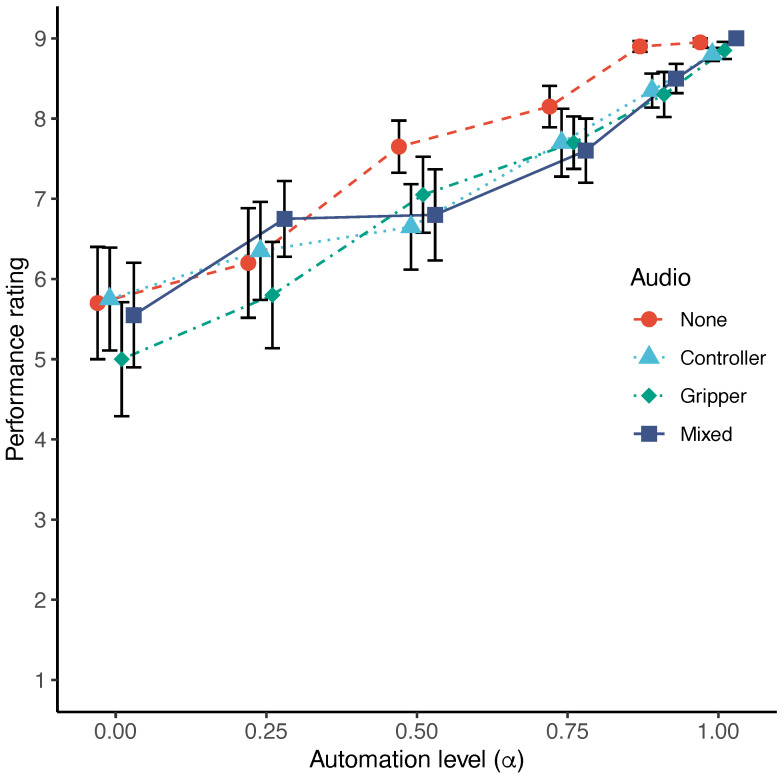
Mean performance ratings at each level of automation under four different auditory feedback conditions. Error bars represent standard errors. Performance rating increases with increasing level of automation.

Figure 7 shows the mean performance ratings at each level of automation under four different auditory feedback conditions. To examine the effect of automation on performance, Friedman’s test was conducted for each auditory feedback condition. The test results show significant differences between the automation levels in all conditions (none: $\chi^{2}\left(5\right)=39.837$, p<0.001; controller: $\chi^{2}\left(5\right)=35.016$, p<0.001; gripper: $\chi^{2}\left(5\right)=43.129$, p<0.001; mixed: $\chi^{2}\left(5\right)=41.050$, p<0.001). To examine the effect of auditory feedback on performance, tests were conducted for each automation level condition. Wilcoxon’s signed rank test was used to test the difference between the two groups, and Bonferroni correction was used for multiple comparisons. The test results indicate that there are no significant differences between the auditory feedback conditions at all automation levels.

All statistical analyses were performed with R version 4.2.1 [32].

## 5. Discussion

In this study, the tracking task was performed in a VR environment with the aim of introducing shared control while maintaining a high SoA by presenting auditory feedback that blurs judgments about the distinction between self and others. Shared control was introduced for the control of the position and rotation of the virtual gripper, and its intensity was manipulated by changing the automation ratio α. Experiments were also conducted under a total of four different auditory feedback conditions to examine the effect of the proposed method, the mixed condition, on the SoA for the gripper’s movements at each intervention intensity. The sound presented in the mixed condition was generated by changing its pitch in conjunction with the combined values of the velocity and angular velocity of the operator’s operation (controller) and those of the controlled object (gripper).

The results of this experiment are in agreement with those of previous research, which showed that SoA decreased as the level of intervention from others increased [31]. However, we did not observe an increase in the SoA that accompanied the increase in task performance, as reported in previous studies [23,24]. We consider that this is because, in this experiment, unlike the fully manual condition in previous studies, the operator’s hand position and posture in the 3D space were aligned with the manipulated object; thus, the operator had already acquired a high SoA before the experiment, which was significantly affected by slight deviations in position or posture.

In this experiment, it was also confirmed that at a moderate level of automation (α=0.50), SoA was significantly higher in the mixed condition than in the none and controller conditions. We consider that this is because, in the none condition, the operator attributed the subject of the object’s movement to others based mainly on their own motion commands and visual feedback, whereas in the mixed condition, auditory feedback including both one’s own and others’ motion information was added to the judgment material, making the subject of the action ambiguous for the operator; then, the operator attributed the subject of the object’s movement to themselves. In other words, presenting the proposed auditory feedback to the operator may have reduced the weight of visual feedback in terms of integrating the sensory signals and maintained a high SoA in relation to the gripper’s motion. This result is consistent with cue integration theory [22], which holds that the generation of SoA is influenced by both internal and external cues, and that external cues influence judgments of the distinction between self and others when the action–feedback relationship is unreliable. In addition, the SoA in the controller and gripper conditions was not significantly different from the SoA in the none condition at the medium automation level. This may be because the sounds presented in the controller and gripper conditions are linked to the operator’s motor commands and visual feedback, respectively, making the discrepancy between the two more apparent. Furthermore, this result confirmed that the effect of the proposed method on the SoA cannot be achieved by simply presenting some sounds.

However, in this experiment, the effectiveness of the proposed method was confirmed only when α was 0.50. For low automation levels (α=0.00,0.25), the position and angle of the controller and gripper did not shift significantly; therefore, the difference in auditory feedback was small and did not affect the SoA. For higher automation levels (α≥0.75), the controller and gripper deviated significantly, and the visual feedback, which is more reliable than other sensory information and has a higher weight of sensory integration [33,34], presented the deviation more prominently; therefore, it can be considered that the difference in sound had no effect on the SoA. This tendency to maintain a high SoA within an acceptable range is similar to the tendency of the results of a previous study that examined the effects of varying the intensity of intentional effort, which is an internal cue, on the SoA [29]. When the influence of intervention from others is extremely high, we consider that the modification of some cues (intentional effort in the previous study, auditory feedback in our study) has little effect; however, within an acceptable range, the modification of cues also increases its reliability. As a result, it is considered that the weight of each cue when integrating the cues changed and the subject of the action was attributed to the self.

Our results may contribute to the operator’s proactive manipulation of the robot while receiving manipulation assistance. However, since this experiment was conducted in a virtual environment to generate the 3D motion of the target object and to facilitate comparison between conditions, it is necessary to verify the effect in the real world using an actual robot. The results of this experiment also suggest that there are limitations in reducing the effect of extreme automation on SoA by modifying some cues. Therefore, in the future, we will design a shared control system in which the robot predicts the operator’s intention (next operation command) to determine the intervention intensity, and we will combine the system with our proposed method to maintain a high SoA. In addition, when environmental sounds around the robot are presented to the operator, the motor drive noise generated by the robot itself may affect the SoA. Therefore, the processing method of the environmental sounds at the site and the type of sounds utilized should be carefully investigated.

## 6. Conclusions

We proposed an auditory feedback method to suppress the diminishing SoA that occurs when shared control is introduced to robot control. We constructed a virtual environment in which a virtual robot gripper was operated using VR devices, and we performed the tracking task to follow a regularly moving object by changing the level of automation for the operation. The experiment was conducted under the following four conditions: the none condition, in which no sound was presented; controller condition, in which sound was presented in conjunction with the movements of the VR controller; gripper condition, in which sound was presented in conjunction with the movements of the virtual gripper; and mixed condition, in which sound was presented in conjunction with the movements of both the controller and the gripper. Our results suggest that our proposed auditory feedback may blur the distinction between self and others, and that operators attribute the subject of the motion of the manipulated object to themselves. This study may contribute to achieving robot operational support through shared control while maintaining a high SoA. In the future, the effectiveness of the proposed method will be verified by remotely operating the actual robot in the real world.

## Data Availability

Not applicable.

## Abbreviations

The following abbreviations are used in this manuscript:

SoA	Sense of Agency
VR	Virtual Reality
HMD	Head-Mounted Display
